# Supplementary material for: Comparative multiomics analysis of cell physiological state after culture in a basket bioreactor
Source: Sci Rep. 2022 Nov 23;12:20161. doi: 10.1038/s41598-022-24687-4 (PMC9686226; doi:10.1038/s41598-022-24687-4)
Supplement: Supplementary file 1 — Supplementary Information 1. [file 41598_2022_24687_MOESM1_ESM.zip › raw data/Transcriptomic raw data/7.AS/2.dasplot/TE1vsTC1/SE/15_TMEM62_NW_023666048.1_101160317_101160454_+@NW_023666048.1_101163405_101163450_+@NW_023666048.1_101174141_101174265_+.pdf]

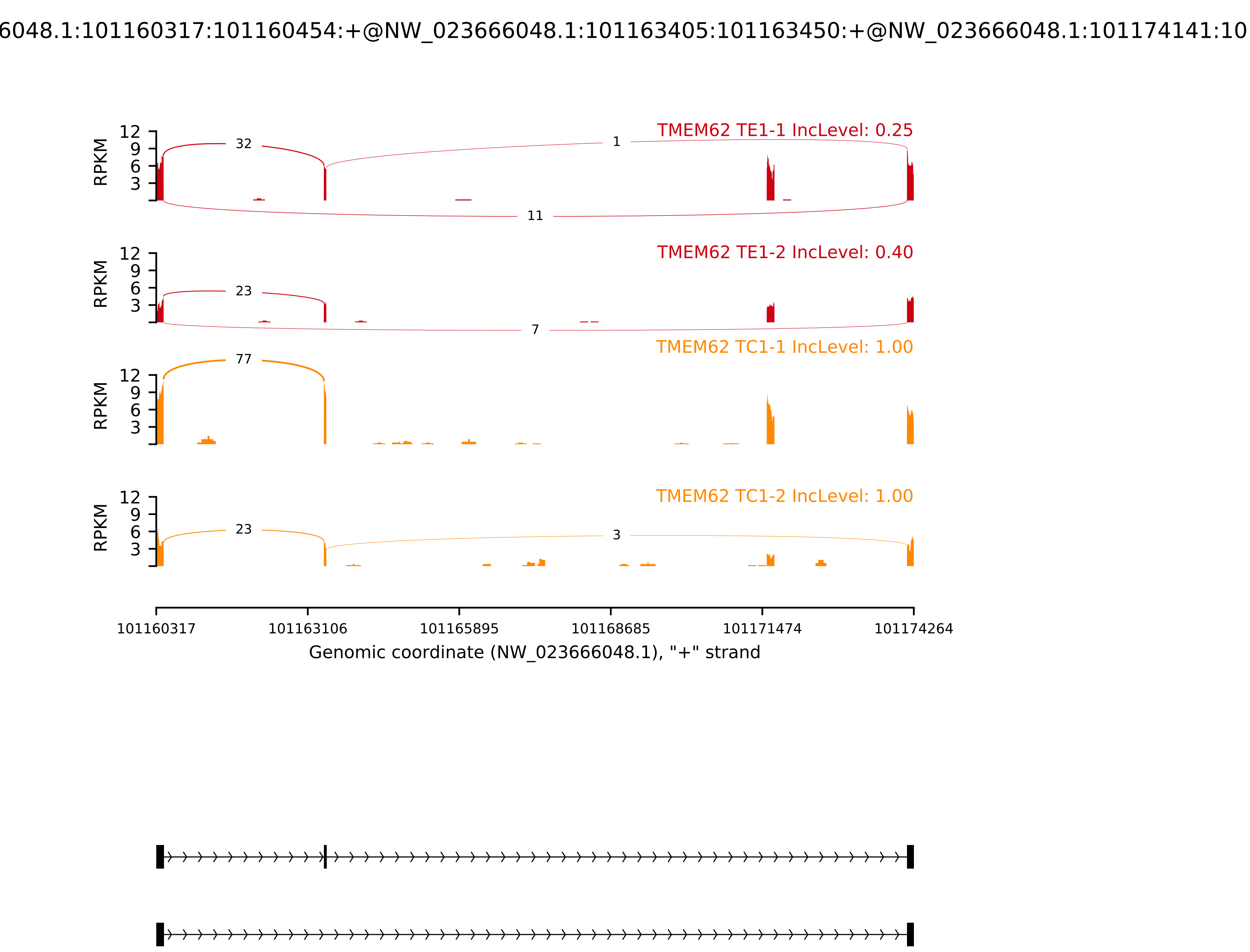
6048.1:101160317:101160454:+@NW_023666048.1:101163405:101163450:+@NW_023666048.1:101174141:10
TMEM62 TE1-1 IncLevel: 0.25
TMEM62 TE1-2 IncLevel: 0.40
TMEM62 TC1-1 IncLevel: 1.00
TMEM62 TC1-2 IncLevel: 1.00
RPKM
12
9
6
3
32
1
11
23
7
77
23
3
101160317
101163106
101165895
101168685
101171474
101174264
Genomic coordinate (NW_023666048.1), "+" strand
